# Drowning in the ripple effect: identifying a syndemic network of health experience (with modifiable health behaviours) using the UK Biobank

**DOI:** 10.1007/s00127-024-02726-x

**Published:** 2024-07-26

**Authors:** Silke Vereeken, Andre Bedendo, Simon Gilbody, Catherine E. Hewit

**Affiliations:** 1 Mental Health and Addiction, Department of Health Sciences, University of York, York, UK; 2 Department of Health Sciences, Faculty of Sciences, University of York, York, UK; 3 Hull York Medical School, York, UK; 4 York Trials Unit, Department of Health Sciences, University of York, York, UK

**Keywords:** SMI, Syndemics, SEM, Health behaviours, Mental health

## Abstract

A Syndemic model of health experience in severe mental illness (SMI) involving modifiable health behaviour contributors has been theorised but has not yet been investigated. Over the next 10 years mental ill-health and suicidal behaviours have been predicted to increase which will decrease health experience and increase hospitalisation and associated costs. This paper investigated a Syndemic model of health experience in people with SMI informed by physical activity levels, exposure to nature, personal resilience levels, drugs related (tobacco smoking and alcohol consumption), and sleep behaviours using UK Biobank data. Results implementing SEM indicate partial evidence for a Syndemic model, with personal resilience being at its centre. Contrary to previous findings, drugs related behaviours did not play an important role in the model. Implementing a Syndemic framework approach to current health care strategies could be beneficial in the development of self-management strategies for people with SMI. This is the first paper using SEM analyses to investigate SMI under the Syndemic theory paradigm.

**Supplementary Information:**

The online version contains supplementary material available at 10.1007/s00127-024-02726-x.

Health experience is generally a combination of physical and mental health experience, influencing quality of life [1]. Suffering from a serious illness impacts the happiness an individual experiences [2]. Major depressive disorder (MDD), bipolar disorder, or psychotic disorder/schizophrenia are commonly combined under the banner of *severe mental illness* (SMI), and has been shown to impact health and health experience negatively to the point of premature and above average mortality rates [3]. SMI in the UK is evidenced to disproportionately affect people in deprived areas compared to least deprived areas, as well as in people over the age of 35 [4]. During the last decennia, global and local health issues have occurred one after the other. The occurrence of illnesses and natural disasters shifting into various epidemics will increase within the next ten years, which is estimated to increase mental ill-health, and suicidal behaviours [5–7]. In turn, it is expected a decrease on individual health experience and increase hospitalisation numbers and associated cost increases. Research aims have therefore shifted from finding contributors to SMI development to finding preventive measures to reduce the prevalence and recurrence of SMI [i.e., by strengthening coping mechanisms in people with SMI [8]]. Currently in mental health care, preventive strategies target risk factors and treat (sub-)clinical manifestations of mental illness to prevent deterioration, multimorbidity, and disability, and promote psychological well-being [8]. However, self-management of mental and physical illnesses for people with SMI through behaviours is important in decreasing hospitalisation rates and improving clinical outcomes [9]. Happiness is positively associated with mental and somatic health [10]. Hence, investigating the relationship between health happiness and health satisfaction, affective states evidenced to affect psychological well-being [11], and engagement in health behaviours in a population of people with SMI could be beneficial in identifying modifiable contributors to individuals’ health experience [2].

Modifiable health behaviours are intentional or unintentional actions that affect health and mortality in individuals, and are a contributor to mental wellbeing [12]. Modifiable health behaviours describe encouraged or supported behaviours that improve health experience with the potential of synergistically improving both quality of life and wellbeing [8]. Engaging in one health behaviour is usually accompanied by engaging in other health behaviours [13], and a synergistic effect of health risk factors on negative (mental) health experience has been connected to engaging in multiple modifiable health risk behaviours concurrently [14]. Several health behaviours representative of current epidemics in Western cultures have also been correlated to SMI and overall health experience; low levels of physical activity [15], drugs related behaviour [16, 17], personal resilience levels [18], exposure/access to green and blue spaces [19], and sleep behaviour [20, 21]. Personal resilience, a set of processes that facilitate recovering from adversity [22], is influenced by individual levels of physical activity [23], felt social support [24] and experiences loneliness [25], and personal strength engagement [22]. Links between these health behaviours and SMI have already been explored individually, but the co-occurrence of them and therefore potential synergistic effect on health and health experience has yet to be investigated.

The Syndemic theory [26] provides an important theoretical background for studying these factors concurrently, as it suggests that a synergistic effect of co-occurring epidemics can exacerbate an individual’s health outcome and/or experience due to Syndemic vulnerability. A Syndemic describes the effect of multiple co-occurring and simultaneously interacting epidemics to exacerbate each epidemic’s effect on individuals [26]. The stress of experiencing these co-occurring contributors can lead to excess burden of disease experience [27], meaning increased stress to treat illness and maintain quality of life, and a decline in health experience due to Syndemic vulnerability. Syndemic vulnerability describes the extent to which individual experiences affect co-occurring social and health problems, morbidity, and mortality because of the eco-psychosocial context of an individual, which again can intensify these problems [28]. Stress, poverty, discrimination, and other forms of social adversity are the primary route through which social factors have been found to contribute to negative health [29–31]. A Syndemic model of these contributors to health experience has been previously theorised [32], but has yet to be tested.

The main aim of this paper is to explore a possible Syndemic model of health experience in people with SMI under the Syndemic theory paradigm, informed by physical activity, drugs related behaviours, personal resilience, exposure to nature, sleep behaviour, and deprivation.

## Methods

### Participants and procedure

This is a secondary data analysis using the UK Biobank’s (UKBB) baseline assessment data collected between 2006 and 2010. The UK Biobank ethical approval research committee approved the use of this data for this study (Ref.11/NW/0382). The UKBB dataset is a community-based cohort study comprising of over 500,000 volunteers aged between 40 and 69 years from across the United Kingdom. Participants have undergone routine, standardised measures, provided blood, urine and saliva samples, given detailed information about themselves and agreed to have their physical health followed. Full details of the study design and data collection processes have been previously published [33, 34].

We used a sample of the UK Biobank based on SMI diagnosis. The population group was created by including data from participants diagnosed with nonaffective psychotic disorder including schizophrenia and schizophrenia-like conditions (ICD-10 disease classes F20-F29; *N* = 155), and/or any bipolar disorder (ICD-10 disease classes F30-31; *N* = 200), and/or any MDD (ICD-10 disease classes F32-39; *N* = 8007).

### Measures

**Fig. 1:**
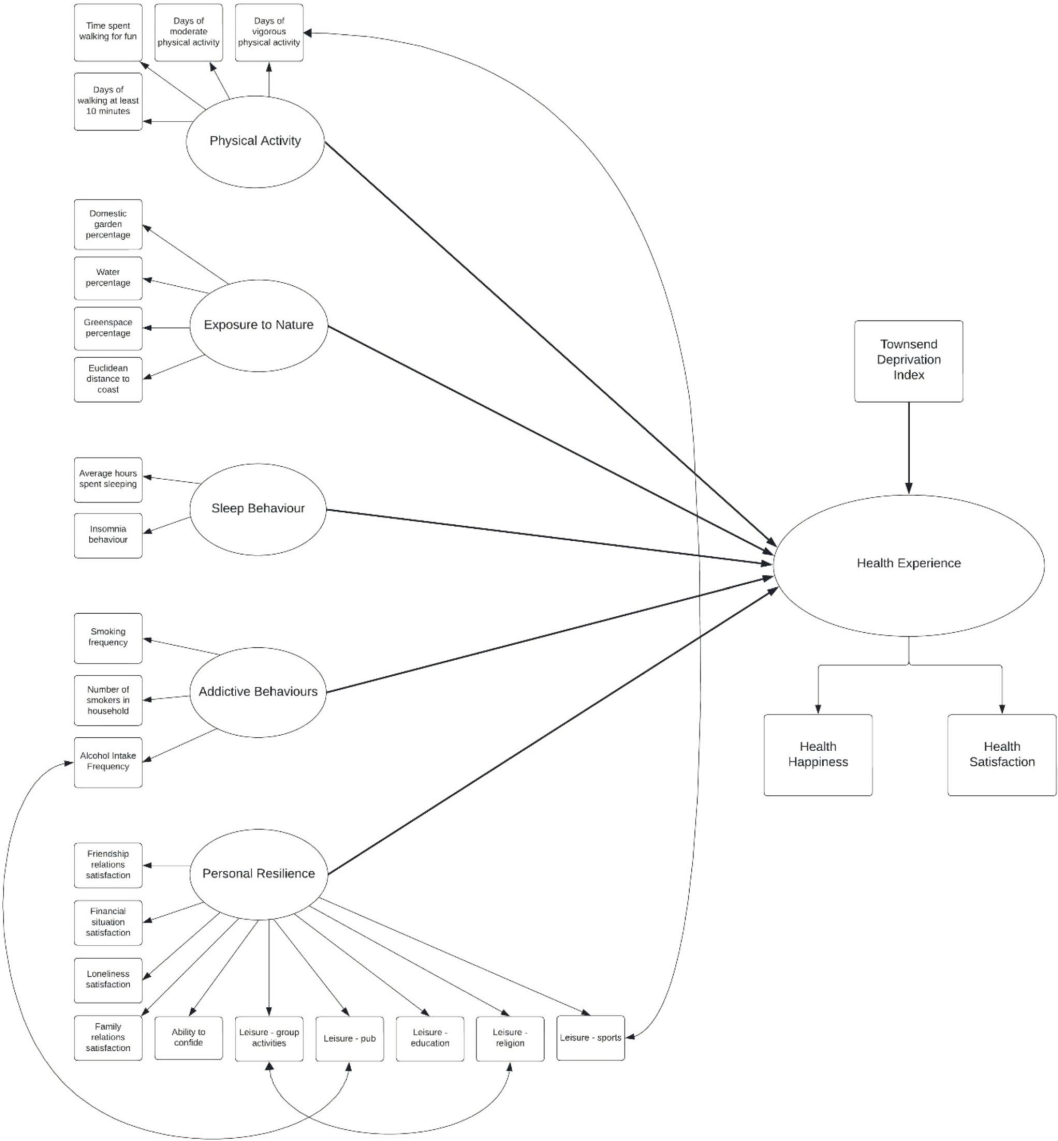
SEM of the presented model

The UK Biobank questions and questionnaires are an accumulation of previously used questionnaires from observational studies, population surveys, and clinical trials to identify appropriate measures of exposure in the different areas [35]. These stem from validated questionnaires, short scales, and clinical interviews. Validity of inclusion was discussed with a wide panel of international experts for each area of interest [35]. Full details on the individual variables (i.e., questions asked to obtain data and response scales) are given in Online Resource 1. To provide a general overview presently, only essential information is listed. The hypothesised model is shown in Fig. 1.

#### Outcome

**Health Experience.** To assess health experience, data from two questions determining general health happiness and general health satisfaction using an 8-point Likert scale (ranging from “prefer not to answer” to “extremely happy”.

#### Modifiable health behaviours

**Physical Activity.** Four questions assessing the level of physical activity the participants on average engage in a typical week were asked, requesting information on time spent doing moderate and vigorous physical activity, and days spent walking 10 or more minutes at a time as well as time spent walking for fun on an average day in the past 4 weeks. Response options were either numerical input between 0 and 7, or on a 9-point Likert scale categorising the time spent doing specific types of physical activity (ranging from “prefer not to answer” to “less than 15 minutes”).

**Drugs Related Behaviours.** Tobacco smoking frequency and number of smokers in household were recorded on a 4-point Likert scale (ranging from “prefer not to answer” to “smokes on most or all days”, and “prefer not to answer” to “yes, more than one household member smokes” respectively). Alcohol intake frequency was recorded on a 6-point Likert scale (ranging from “prefer not answer” to “daily or almost daily”).

**Personal Resilience.** We considered 9 questions addressing social support, friendship relations satisfaction, family relations satisfaction, financial security, and ability to confide [36], experienced loneliness [25], and leisure activities [22]. Responses were recorded on an 8-point Likert scale (ranging from “prefer not to answer” to “extremely happy”) for part of the questions, a 3-point Likert scale (loneliness satisfaction), or as Yes/No (experienced loneliness).

**Exposure to Nature.** Four variables assessed the participants’ proximity levels to nature. We accessed data on green spaces (land coverage estimates for domestic gardens and natural vs. built environments within 1000 m of the participant’s home location) and blue spaces (coastal proximity, access to bodies of water within 1000 m of the participant’s home location). The data linkage for this category was done by the European Centre for Environment and Human Health (University of Exeter Medical School) and for this project’s purposes includes greenspace estimates at 1000 m home location buffers, land coverage estimates for domestic gardens and water at 1000 m home location buffers, land coverage estimates for the ‘natural environment’ compared to the ‘built environment’ (greenspace percentage) at 1000 m home location buffer, and distance (Euclidean) from home location to the coast measured in Kilometres.

**Sleep Behaviour.** Average hours spent sleeping, requiring a numerical input between 1 and 23, and insomnia behaviour/sleeplessness on a 4-point Likert scale ranging from “prefer not to answer” to “usually” were included.

#### Syndemic contributors

**Townsend Deprivation Index.** Additionally, the Townsend Deprivation Index (TDI) was included in the model as a Syndemic contributor to health experience. This is based on the preceding national census output areas and corresponds to the participant’s postcode as baseline measurement. This variable acts as a proxy for the measure of SES and poverty [37].

**Ethnicity**. Ethnic background determined by a self-report touchscreen questionnaire was included as a possible Syndemic contributor to health experience. This acts as a proxy for a measure of possible discrimination and other forms of social adversity [38, 39].

### Data analysis

Structural equation modelling (SEM) was used to investigate the relationships between the modifiable health behaviours outlined above and how they relate to health experience. Relevant summary statistics were initially assessed using mean and standard deviations for continuous data and counts and percentages for categorical data. Spearman correlation coefficients were calculated to identify any multicollinearity that might reduce model validity and strength if kept [40]. The analysis used standard indices to evaluate model fit; the comparative fit index (CFI), the Tucker-Lewis index (TLI), and the root mean square error (RMSEA). Threshold scores of 0.90 or higher for the CFI and TLI, as well as a RMSEA value of 0.08 or lower were used as indication of a good model fit (Finch, 2020). All relevant statistics and model parameters of interest including confidence intervals are reported. Analyses were conducted in R (version 4.0.5) using RStudio and *lavaan* package [41, 42].

We report the relevant summary statistics for each analysis (i.e., the averages and error surrounding measurements of interest and the composition and size of the sample) along with the model parameters of interest and the associated 95% confidence intervals of these estimates.

## Results

### Descriptive statistics and correlations

Out of the 500,000 participants in the UKBB database, a total of 8,014 participants had an SMI diagnosis and provided data on the variables of interest. Only data from participants who responded to all questions were included. On average, the participants were 55.61 years old (*SD* = 7.71 years), 70% were female and the majority were white (96.72%; Table 1). Table 1 gives an overview of all averages and standard deviations of the variables used in this model.

**Table 1:** Means and standard deviations for all variables included in the model, grouped by SMI diagnosis

	Depression (*N* = 8007; 99.99%)	Bipolar Disorder (*N* = 200; 2.5%)	Schizophrenia or Psychosis Disorder (*N* = 155; 1.9%)
*Age*			
− Mean (SD)	55.6 (7.7)	55.0 (8.1)	54.6 (8.2)
*Sex*			
− Male	2404 (30%)	85 (42.5%)	57 (36.8%)
− Female	5603 (70%)	115 (57.5%)	98 (63.2%)
*Ethnicity* − White − Asian or Asian British − Black or Black British − Mixed − Other	7744 (96.7%) 71 (0.9%) 57 (0.7%) 55 (0.7%) 80 (1.0%)	185 (92.5%) 4 (2.0%) 2 (1.0%) 3 (1.5%) 6 (3.0%)	142 (91.6%) 1 (0.7%) 2 (1.3%) 3 (1.9%) 7 (4.5%)
*Townsend Deprivation Index* − Mean (SD)	-1.35 (2.8)	-0.5 (3.2)	-0.17 (3.5)
*Amount of days/week doing moderate physical activity (> 10 min)*			
− Mean (SD)	3.7 (2.3)	3.6 (2.4)	3.4 (2.5)
*Amount of days/week doing vigorous physical activity (> 10 min)*			
− Mean (SD)	1.8 (1.8)	1.9 (2.0)	1.6 (2.0)
*Amount of days/week going for a walk (> 10 min)*			
− Mean (SD)	5.6 (1.7)	5.9 (1.6)	5.8 (1.7)
*Time spent walking for pleasure*			
− Less than 15 min − Between 15 and 30 min − Between 30 min and 1 h − Between 1 and 1.5 h − Between 1.5 and 2 h − Between 2 and 3 h − Over 3 h	136 (2.0%) 1525 (19.0%) 2970 (37.1%) 1483 (18.4%) 791 (9.9%) 545 (6.7%) 557 (6.9%)	2 (1.0%) 52 (26.0%) 75 (37.5%) 29 (14.5%) 23 (11.5%) 7 (3.5%) 12 (6.0%)	7 (4.5%) 41 (26.5%) 53 (34.2%) 18 (11.6%) 19 (12.3%) 11 (7.1%) 6 (3.9)
*Sleep duration*			
− Mean (SD)	7.2 (1.1)	7.6 (1.5)	7.5 (1.4)
*Sleeplessness/Insomnia*			
− Never/Rarely	1471 (18.4%)	40 (20%)	41 (26.4%)
− Sometimes	3765 (47%)	98 (49%)	65 (42%)
− Usually	2771 (34.6%)	62 (31%)	49 (31.6%)
*Current Tobacco Smoking*			
− No	7773 (97.1%)	186(93%)	149(96.1%)
− Only occasionally	234 (2.9%)	14 (7%)	6 (3.9%)
− Yes, on most or all days	0 (0.0%)	0 (0.0%)	0 (0.0%)
*Smokers in Household*			
− No Household Member Smokes	7229 (90.3%)	178 (89%)	135 (87.1%)
− 1 Household Member Smokes	673 (8.4%)	18 (9%)	16 (10.3%)
− More than 1 Household Member Smokes	105 (1.3%)	4 (2%)	4 (2.6%)
*Frequency of drinking alcohol*			
− Never	513 (6.4%)	24 (12%)	22 (14.2%)
− Special Occasions Only	907 (11.3%)	31 (15.5%)	29 (18.7%)
− 1 to 3 Times a Month	1045 (13.1%)	24 (12%)	22 (14.2%)
− Once or Twice a Week	1923 (24.0%)	31 (15.5%)	26 (16.8%)
− 3 or 4 Times a Week − Daily or Almost Daily	1915 (23.9%) 1704 (21.3%)	46 (23%) 44 (22%)	22 (14.2%) 34 (21.9%)
*Greenspace Percentage (1000 m)*			
− Mean (SD)	42.6 (22.2)	37.9 (21.4)	36.8 (19.5)
*Water Percentage (1000 m)*			
− Mean (SD)	1.3 (2.6)	1.3 (2.9)	1.3 (2.9)
*Euclidean Distance to Coast*			
− Mean (SD)	45.7(26.3)	46.6 (25.0)	46.2 (24.5)
*Domestic Garden Percentage (1000 m)*			
− Mean (SD)	26.5 (11.7)	28.3 (11.3)	28.3 (10.8)
*Family-relationship Satisfaction*			
− Prefer not to say	1196 (14.9%)	19 (9.5%)	12 (7.7%)
− Extremely Unhappy	68 (0.9%)	6 (3.0%)	7 (4.5%)
− Very Unhappy	73 (0.9%)	4 (2.0%)	5 (3.2%)
− Moderately Unhappy	158 (2.0%)	6 (3.0%)	6 (3.8%)
− Moderately Happy	621 (7.8%)	14 (7.0%)	13 (8.5%)
− Very Happy	2718 (33.9%)	87 (43.5%)	57 (36.8%)
− Extremely Happy	3173 (39.6%)	64 (32.0%)	55 (35.5%)
*Financial Situation Satisfaction*			
− Prefer not to say	575 (7.2%)	13 (6.5%)	8 (5.1%)
− Extremely Unhappy	23 (0.3%)	2 (1%)	4 (2.6%)
− Very Unhappy	161 (2.0%)	12 (6%)	11 (7.1%)
− Moderately Unhappy	266 (3.3%)	7 (3.5%)	6 (3.9%)
− Moderately Happy	793 (9.9%)	18 (9.0%)	20 (12.9%)
− Very Happy	6357 (45.7%)	94 (47.0%)	71 (45.8%)
− Extremely Happy	2532 (31.6%)	54 (27.0%)	35 (22.6%)
*Friendships Satisfaction*			
− Prefer not to say	915 (11.4%)	18 (9.0%)	10 (6.5%)
− Extremely Unhappy	81 (1.0%)	6 (3.0%)	11 (7.1%)
− Very Unhappy	21 (0.3%)	2 (1.0%)	3 (1.8%)
− Moderately Unhappy	51 (0.6%)	4 (2.0%)	2 (1.3%)
− Moderately Happy	415 (5.2%)	18 (9.0%)	10 (6.5%)
− Very Happy	2805 (35.0%)	79 (39.5%)	59 (38.1%)
− Extremely Happy	3719 (46.5%)	73 (36.5%)	60 (38.7%)
*Ability to Confide in Others*			
− Never or Almost Never	945 (11.8%)	29 (14.5%)	27 (17.4%)
− Once Every Few Months	491 (6.1%)	15 (7.5%)	10 (6.5%)
− About Once a Month	584 (7.3%)	14 (7.0%)	12 (7.7%)
− About Once a Week	1080 (13.4%)	32 (16.0%)	23 (14.8%)
− 2–4 Times a Week	1049 (13.1%)	25 (12.5%)	21 (13.6%)
− Almost Daily	3858 (48.2%)	85 (42.5%)	62 (40%)
*Loneliness/Isolation*			
− No	5743(71.7%)	125 (62.5%)	95 (61.3%)
− Yes	2264 (28.3%)	75 (37.5%)	60 (38.7%)
*Leisure Activity - Sports*			
− No	5465(68.3%)	143 (71.5%)	124 (80.0%)
− Yes	2542 (31.7%)	57 (28.5%)	31 (20.0%)
*Leisure Activity - Pub*			
− No	6350 (79.3%)	158 (79.0%)	126 (81.3%)
− Yes	1657 (20.7%)	42 (21%)	29 (18.7%)
*Leisure Activity - Education*			
− No	7090 (88.5%)	171 (85.5%)	127 (81.9%)
− Yes	917 (11.5%)	29 (14.5%)	28 (18.1%)
*Leisure Activity - Religion*			
− No	6655 (83.1%)	154 (77.0%)	117 (75.5%)
− Yes	1352 (16.9%)	46 (23.0%)	38 (24.5%)
*Leisure Activity - Group Activity*			
− No	4832 (60.3%)	110 (55.0%)	80 (51.6%)
− Yes	3175 (39.7%)	90 (45.0%)	75 (48.4%)
*General Happiness with own health*			
− Do Not Know/Prefer Not to Answer	33 (0.1%)	11 (0.3%)	22 (0.3%)
− Extremely Unhappy	91 (0.4%)	30 (0.8%)	130 (1.7%)
− Very Unhappy	282 (1.1%)	86 (2.3%)	285 (3.8%)
− Moderately Unhappy	1555 (6.0%)	377 (10.1%)	989 (13.3%)
− Moderately Happy	10,286 (39.6%)	1723 (46.2%)	3333 (44.7%)
− Very Happy	11,057 (42.5%)	1246 (33.4%)	2232 (30.0%)
− Extremely Happy	2692 (10.4%)	260 (7.0%)	458 (6.1%)
*Health Satisfaction*			
− Do Not Know/Prefer Not to Answer	42 (0.2%)	10 (0.3%)	18 (0.2%)
− Extremely Unhappy	42 (0.2%)	13 (0.3%)	66 (0.9%)
− Very Unhappy	172 (0.7%)	67 (1.8%)	209 (2.8%)
− Moderately Unhappy	1421 (5.5%)	313 (8.4%)	852 (11.4%)
− Moderately Happy	11,657 (44.8%)	1860 (49.8%)	3759 (50.5%)
− Very Happy	10,897 (41.9%)	1308 (35.0%)	2247 (30.2%)
− Extremely Happy	1765 (6.8%)	162 (4.3%)	298 (4.0%)
*Health Score* − Mean (SD)	-0.21 (0.8)	− 0.015 (0.8)	-0.09 (0.8)

### Syndemic model

Spearman correlation coefficients indicated a high correlation between the two Syndemic contributors, ethnicity and TDI. As TDI is a continuous variable based on externally verified census methods, and ethnicity is a categorical self-input variable, we elected to eliminate ethnicity from the model to improve model stability. Primary analyses indicated high correlations between several variables (days engaging in vigorous physical activity and practising a sport during leisure activities; engaging in religious activities in leisure time and engaging in group activities in leisure time; and alcohol intake frequency and pub visits in leisure time), which were allowed to be estimated in the model. The measurement model analysis confirmed that the selected variables show appropriate fits (Leisure time – pub and Leisure time – education for the *Personal Resilience* construct, and smoke frequency for the *Drugs Related Behaviours* construct). Sensitivity analyses on the effect of removing these contributors from the model resulted in comparable model indices, so to provide a broader insight into the model, we elected to keep the contributors in the final SEM.

The results of the SEM indicated a reasonably good fit of the tested model. Although χ² was significant (χ²= 6035.766, *p* < 0.001, df = 281), the TLI and CFI indicators demonstrate a reasonably good model fit (TLI = 0.786, CFI = 0.815). The RMSEA indicated a very good fit of the model (RMSEA = 0.051). This implies that the model provides a reasonably good fit to the data, however, the model could probably be improved. A closer investigation of the individual relationships within the model for future iterations is therefore necessary.

**Table 2:** Measurement model specifying how the indicators (measured variables) correspond to the latent constructs^1^

Variables	Unstandardized Coefficient	Standardized Coefficient (β)*	*R*²**	*p* Value
Physical Activity				
Time spent walking for Fun	1.000	0.1	0.012	
Amount of days/week going for a walk	4.659	0.39	0.151	< .001
Amount of days/week doing moderate physical activity	12.340	0.78	0.607	< .001
Amount of days/week doing vigorous physical activity	7.563	0.61	0.374	< .001
Drugs Related Behaviours				
Alcohol intake frequency	1.000	0.07	0.021	
Frequency of Smoking	1.170	0.77	< 0.001	0.590
Amount of Smokers in household	0.422	0.13	0.002	0.004
Personal Resilience				
Leisure – Sports	1.00	0.09	0.109	
Leisure – Education	0.124	0.02	< 0.001	0.212
Leisure – Group Activities	1.150	0.1	0.009	< .001
Leisure – Pub	0.150	0.3	0.052	0.208
Leisure – Religion	0.159	0.02	0.309	0.093
Friendship Satisfaction	13.664	0.64	0.401	< .001
Ability to Confide in Others	18.769	0.46	0.204	< .001
Financial Situation Satisfaction Loneliness/Isolation Family Relationships Satisfaction	9.451 -4.777 15.466	0.4 -0.46 0.63	0.166 0.210 0.399	< .001 < .001 < .001
Exposure to Nature				
Euclidean Distance to the Coast	1.000	0.09	0.187	
Water Percentage	-0.157	-0.13	0.015	< .001
Greenspace Percentage	-5.001	-0.51	0.544	< .001
Domestic Garden Percentage	8.287	1.59	0.566	< .001
Sleep Behaviour				
Number of Hours spent Sleeping	1.000	0.35	0.123	
Sleeplessness/Insomnia Behaviour	-1.375	-0.76	0.581	< .001
Health Experience				
General Happiness about own Health	1.000	0.65	0.370	
Personal Health Satisfaction	1.126	0.76	0.629	< .001

^1^The exogenous latent constructs are physical activity, personal resilience, exposure to Nature, Sleep Behaviour, and drug related Behaviour. The endogenous construct is Health experience

The regression results depicting direct effects for all latent factors are outlined in Table 2. Within the latent factors of personal resilience, exposure to nature, physical activity level, and sleep behaviour the selected contributors were found to be significantly related to each other with few exceptions. In the latent factor of personal resilience, education pursued in leisure time (*p* = 0.212), engaging in religious activities in leisure time (*p* = 0.093), and going to the pub in leisure time (*p* = 0.205) were not found to be significantly related to the other representatives. Similarly, for the latent factor drugs related behaviour, the frequency of smoking was not significantly related to alcohol intake (*p* = 0.590), whereas the amount of smokers in the household was (*p* = 0.004).

**Table 3:** SEM regression analysis

Variables	Unstandardized Coefficient	Standardized Coefficient (β)*	*p* Value	95% CI
Health Experience				
Physical Activity	0.784	0.18	< 0.001	-0.901, 1.112
Drugs Related Behaviours	0.068	0.01	0.742	2.074, 6.656
Personal Resilience	6.069	0.41	< 0.001	-14.543, -0.117
Exposure to Nature	-0.004	-0.02	0.023	-0.006, 0.032
Sleep behaviour	0.282	0.18	< 0.001	0.241, 1.288
Townsend Deprivation Index	-0.013	-0.06	< 0.001	-0.014, -0.002
Personal Resilience – Religion				
Personal Resilience – Group Activities	0.425	0.55	< 0.001	0.407, 0.442
Personal Resilience – Sports				
Physical Activity – Amount of days/week doing vigorous physical activity	0.076	0.3	< 0.001	0.070, 0.081
Personal Resilience – Pub				
Drugs Related Behaviours – Alcohol Intake frequency	0.061	0.23	< 0.001	0.056, 0.66

Table 3 gives an overview of all main effects. All latent constructs predicted health experience, apart from drugs related behaviours (*p* = 0.742). This implies that the latent variables for physical activity, exposure to nature, personal resilience, and sleep behaviour, as well as the measure for TDI all predict health experience. The higher the level of physical activity, personal resilience, and good sleep behaviours, the higher the health experience. Equally, the lower TDI, the higher health experience. Contrary to theoretical evidence, a lower exposure to nature tends to predict a higher health experience. Drugs related behaviours seem to not be directly related to health experience in the context of this data and model.

**Fig. 2:**
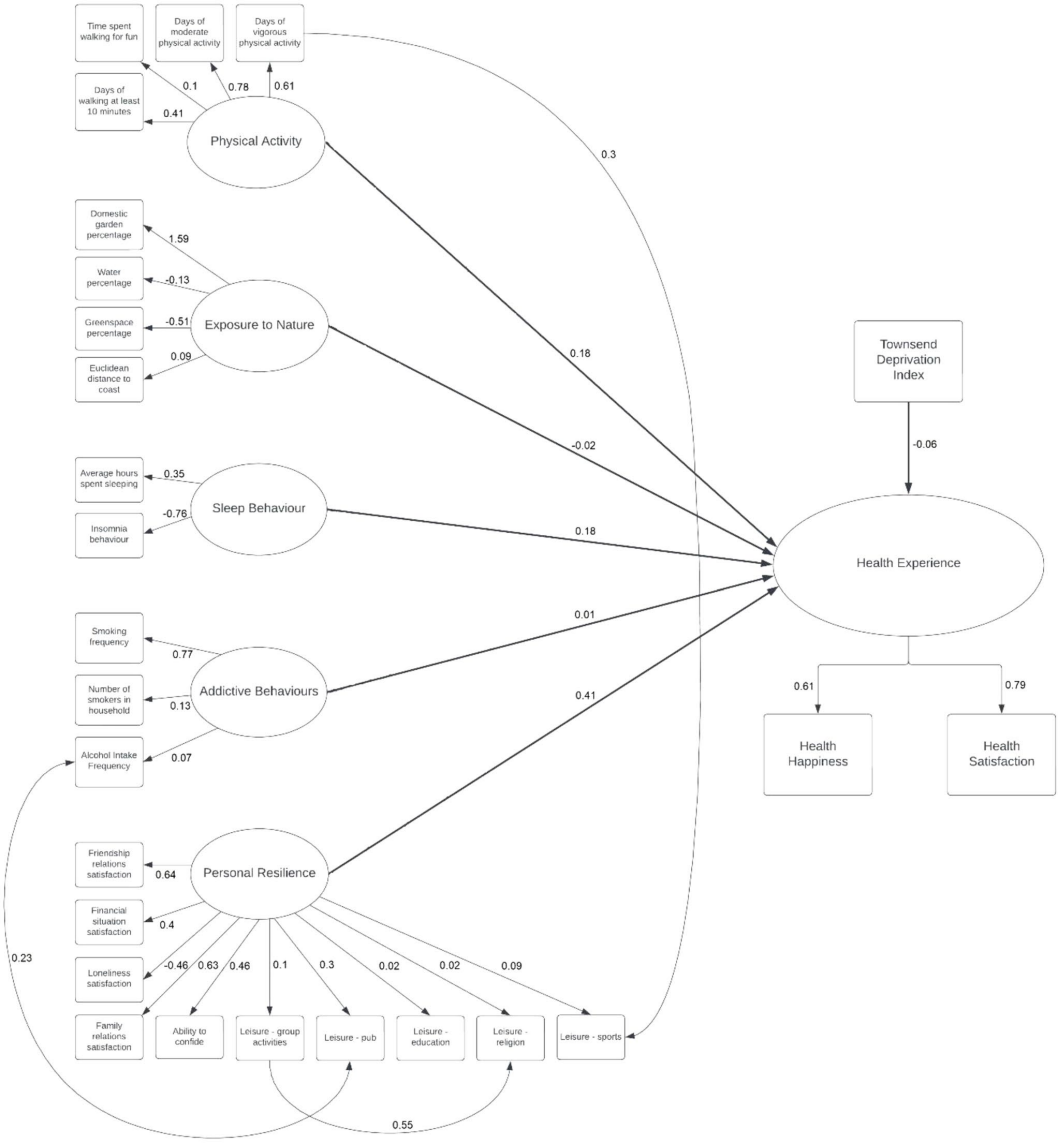
Final SEM of the investigated Syndemic Model showing standardised coefficients. Standard path coefficients between the latent variables have been omitted for readability

Figure 2 shows relationships between the variables of the model with effects and standardised path coefficients. The standardised path coefficients between the latent variables have been excluded to improve readability.

Table 4 shows the correlations between the latent factors of health behaviours. The latent factors of exposure to nature, physical activity, and sleep behaviour were significantly related to personal resilience (*p ≤* 0.001). This implies that individual levels of personal resilience affect individual levels of exposure to nature, physical activity, and sleep behaviour. Beyond these significant relationships, no other latent factors showed significant interrelatedness.

**Table 4:** Correlation between the latent factors

Covariances	Unstandardized Coefficient	Standardized Coefficient (β)*	*p* Value	95% CI
Personal Resilience				
Exposure to Nature	0.003	-0.029	0.001	-0.060, 0.001
Physical Activity	0.001	0.110	< 0.001	0.076, 0.143
Sleep Behaviour	0.004	0.223	< 0.001	0.174, 0.271
Drugs Related Behaviours	0.000	0.723	0.576	0.581, 0.864
Exposure to Nature				
Physical Activity	-0.003	-0.014	0.142	-0.045, 0.017
Sleep Behaviour	-0.008	0.021	0.162	-0.013, 0.055
Drugs Related Behaviour	-0.007	-0.157	0.598	-0.319, 0.006
Physical Activity				
Sleep Behaviour	0.001	0.014	0.484	-0.024, 0.052
Drugs Related Behaviour	-0.000	0.184	0.964	0.013, 0.354
Sleep Behaviour				
Drugs Related Behaviours	0.001	-0.032	0.538	-0.221, 0.156

## Discussion

The present study aimed to explore a potential Syndemic model of health experience in people with SMI informed by physical activity levels, drugs related behaviours, personal resilience, exposure to nature, sleep behaviour, and TDI. SEM analyses partially supported the hypothesised Syndemic relationship between known contributors (exposure to nature, physical activity levels, psychological resilience, and sleep behaviour) and health experience [43–46]. Contrary to theoretical evidence, the relationship between drugs related behaviours and health experience did not fit into this novel Syndemic model [47].

### Theoretical implications

In support of previous publications, most investigated contributors significantly affect health experience. Evidence for the existence of a *Syndemic* model of intercorrelated contributors that affect health experience has not been published yet; this is the first study about a Syndemic model of health experience fed by modifiable health behaviours as contributors. Though some model fit indices did not reach the desired threshold of 0.9 [48], the model aids in developing the theory of Syndemics in health experience in an SMI population. Ample theoretical evidence from both the theory of Syndemics [26] as well as health behaviour contributors in mental health-focused research (references 15–25) has thoroughly informed the development of both this model and theory. This study provides evidence in support of behaviours contributing to personal resilience being intercorrelated to physical activity, sleep behaviour and exposure to nature, as well as being related to health experience in people with SMI; which is in support of a Syndemic model of health experience and resilience-related health behaviours in an SMI population.

Additionally, this is the first study investigating the existence of a Syndemic model using SEM as an analysis tool. Though previously suggested as the way forward in investigating Syndemic models [49], recent research into Syndemic models has not yet implemented SEM to analyse the Syndemic relationships between contributors in one analytical model rather than using individual regression paths between outcome and possible contributors.

Though ample evidence supports drugs related behaviours to contribute to health experience [50, 51], they did not come forward as a significant predictor in our model. Despite suggesting that drugs related behaviours may play a different role other than the other modifiable health behaviours tested (i.e., physical activity, sleep behaviour, and personal resilience), further studies are still necessary. For example, drugs related behaviour may operate on as mediator or moderator of the effects of other behaviours on the health experience. In addition, the variables available to construct the drugs related behaviours latent variable did not include any dependence-related measure. Therefore, we were not able to test whether adding substance-related problems or addiction diagnosis would change the observed results. There is therefore scope for investigating which model of Syndemic contributors to health experience drugs related behaviours fit into; the current Syndemic model is focused on modifiable health behaviours, but drugs related behaviours measured this way may be more closely correlated to a different Syndemic layer health experience.

### Limitations and future research

UK Biobank have stated that the sample collected for the dataset shows evidence of a “healthy volunteer” bias and is therefore not representative of the general population on information collected for lifestyle, sociodemographic, health-related, nor physical [34]. Additionally, the sample is predominantly female (70%) and the mean age of 55 years suggests an older age group than the population mean [52]. Due to the distribution of SMI within the sample, and 99.99% of the sample reporting an MDD diagnosis, the findings can be mainly attributed to a population suffering from depression symptoms. Furthermore, the distribution of SMI diagnoses of bipolar disorder and schizophrenic spectrum disorder is below the estimated average in Britain [53]. Therefore, these results are not generalisable to the wider population, and only to a population with SMI diagnoses.

Furthermore, a limit of the otherwise very thorough and vast UKBB dataset means that exposure to nature can only accurately be determined by measuring the proximity of participants to green or blue spaces. Arguably, proximity to green and blue spaces does not mean that someone is exposed to natural settings more often as well; participants living in areas that are deprived of green and blue spaces might still make the effort of seeking exposure to natural environments which is however not recorded in the dataset. Including questions that specifically measure seeking exposure to natural environments like blue and green spaces could be a useful addition to the already substantial UKBB dataset. Comparably, drugs related behaviour measurements were limited in their depth and breadth in the UKBB; probably due to the UK’s successful anti-smoking campaigns [54], there is only a small percentage of smokers in the dataset, which makes measuring the extent of the effect of these behaviours less informative.

## Conclusion

The results of these analyses provide evidence for the existence of a Syndemic network of health behaviours as contributors to health experience in an SMI population. Previously established contributors to detrimental health experience in people with SMI not just singularly affect health experience, but also interact and syndemically exacerbate health experience. Health care policies and practices should therefore move from a strategy of challenging singular contributors one at a time to tackling multiple contributors simultaneously. Implementing policies and strategies using the Syndemic framework approach could therefore improve the effectiveness of current health care strategies and provide better health care options for a population with SMI, including evidence-based self-management strategies. Future research into the Syndemic network of contributors to health experience and how health care strategies could improve it would be beneficial for populations nationally and ultimately, globally.

## Electronic supplementary material

Below is the link to the electronic supplementary material.

Supplementary Material 1

## Data Availability

This research has been conducted using the UK Biobank Resource under Application Number 91042.
