# Supplementary material for: Drowning in the ripple effect: identifying a syndemic network of health experience (with modifiable health behaviours) using the UK Biobank
Source: Soc Psychiatry Psychiatr Epidemiol. 2024 Jul 26;60(1):235–47. doi: 10.1007/s00127-024-02726-x (PMC11790710; doi:10.1007/s00127-024-02726-x)
Supplement: Supplementary file 1 — Supplementary Material 1 [file 127_2024_2726_MOESM1_ESM.docx]

Drowning in the Ripple Effect: Identifying a Syndemic Network of Health Experience (with Modifiable Health Behaviours) using the UK Biobank

Silke Vereeken*, Mental Health and Addiction, Department of Health Sciences, University of York, UK (Orcid number 0000-0003-1432-8712)

silke.vereeken@york.ac.uk

Andre Bedendo, Department of Health Sciences, Faculty of Sciences, University of York, UK

Simon Gilbody, Mental Health and Addiction, Department of Health Sciences, University of York, UK; Hull York Medical School, York

Catherine E Hewit, York Trials Unit, Department of Health Sciences, University of York, UK

SUPPLEMENTARY INFORMATION

*Supplement 1. Detailed description of selected variables for the model*

**Addictive Behaviours.** Addictive behaviours were measured focusing on tobacco and alcohol addiction indicators.

*Tobacco addiction/exposure.* To assess the level of tobacco smoking addiction and tobacco smoke exposure, the questions “Do you smoke tobacco now?” and “Does anyone in your household smoke?” were asked. Participants had to respond on a 4-point Likert scale ranging from “Prefer not to answer”, “No”, and “Only Occasionally” to “Yes, on most or all days” for the current tobacco smoking status question, and “Prefer not to answer”, “No”, and “Yes, one household member smokes” to “Yes, more than one household member smokes”.

*Alcohol addiction.* To assess the level of alcohol addiction and consumption, the question “How often do you have a drink containing alcohol?” was asked. Participants had to respond on a 6-point Likert scale using “Prefer not to answer”, “Never”, and “Monthly or less”, “2 to 4 times a month”, “2 to 3 times a week”, and “4 or more times a week” as options. Furthermore, the derived variable “Alcohol Drinker Status” was used, with the response options of “Prefer not to answer”, “Never”, “Previous”, and “Current”. Additionally, the datapoint of “Amount of Alcohol drunk on a typical drinking day” was included. Response options were categorised into “1 or 2”, “3 or 4”, “5 or 6”, “7, 8 or 9”, “10 or more”, and “prefer not to answer”.

**Physical Activity.** To assess the level of physical activity the participants engaged in, the questions “In a typical week, on how many days did you do 10 minutes or more of moderate physical activities like carrying light loads, cycling at a normal pace?”, “In a typical week, on how many days did you do 10 minutes or more of vigorous physical activities? (These are activities that make you sweat or breathe hard such as fast cycling, aerobics, heavy lifting)”, “In a typical week, on how many days did you walk for at least 10 minutes at a time?”, and “Each time you went walking for pleasure, about how long did you spend doing it?” were asked. The questions regarding moderate physical activity, vigorous physical activity, and days spent walking 10 or more minutes at a time could be answered by a number from 0 to 7, or the options “Do not know” or “Prefer not to answer”. The options to the question on how much time was spent walking for fun were “Prefer not to answer”, “Do not know”, “Over 3 hours”, “Between 2 and 3 hours”, “Between 1.5 and 2 hours”, “Between 1 and 1.5 hours”, “Between 30 minutes and 1 hour”, “Between 15 and 30 minutes”, and “Less than 15 minutes”. Additionally, the question “Each time you went walking for pleasure, about how long did you spend doing it?” was included to assess duration of light physical activity. Response options were categorised into “Less than 15 minutes”, “Between 15 and 30 minutes”, “Between 30 minutes and 1 hour”, “Between 1 and 1.5 hours”, “Between 1.5 and 2 hours”, “Between 2 and 3 hours”, “Over 3 hours”, “Do not know” and “Prefer not to answer”.

**Exposure to Nature.** To assess the participants’ exposure levels to nature (green and blue spaces), several variables measuring greenspace and coastal proximity from the “Greenspace and coastal proximity (151)” category using the participants’ home location postal code and grid references were selected. The data linkage for this category was done by the European Centre for Environment and Human Health (University of Exeter Medical School) and for this project’s purposes includes greenspace estimates at 1000m home location buffers, land coverage estimates for domestic gardens and water at 1000m home location buffers, land coverage estimates for the ‘natural environment’ compared to the ‘built environment’ (greenspace percentage) at 1000m home location buffer, and distance (Euclidean) from home location to the coast measured in Kilometres.

Additionally, two data points were included to assess the time participants would spend outdoors that could indicate time exposed to nature. These were *Time Spent Outdoors in Summer* and *Time Spent Outdoors in Winter*. The questions asked were “In a typical *day* in winter [summer], how many hours do you spend outdoors?”. Numerical input was requested, and responses below 0 and above 24 were rejected. Participants had to confirm their input if numbers above 10 were submitted.

**Sleep Behaviour.** To assess sleep behaviours in participants, the data from two questions were used: “About how many hours of sleep do you get in every 24 hours? (please include naps)” allowed responding with any number between and including 1 and 23, and a prompt was given to confirm if the number was below 3 or above 12. “Do not know” and “Prefer not to answer” were given as alternative response options. To assess insomniac tendencies, “Do you have trouble falling asleep at night or do you wake up in the middle of the night?“ was asked. Response options were “Prefer not to answer”, “Usually”, “Sometimes”, and “Never/rarely”.

**Personal Resilience.** To assess levels of personal resilience, known contributors to personal resilience were used as proxy determinants. To assess felt social support, variables measuring friendship relations satisfaction, family relations satisfaction, and ability to confide (Sippel et al., 2015) were assembled. To assess experienced loneliness (Gerino et al., 2017), a variable measuring loneliness satisfaction was assembled. To assess levels of physical activity related to improved personal resilience (Ozkara et al., 2016) and other forms of leisure activities improving social-connection related to personal resilience (Scarf et al., 2016), variables measuring leisure activities and hobbies were assembled.

*Friendship Relations Satisfaction.* To assess the level of friendship relation satisfaction, the question “In general, how satisfied are you with your Friendships?” was asked. An 8-point Likert scale ranging from “Extremely unhappy” to “Extremely happy” and including “Do not know” and “Prefer not to answer” made up the response choices.

*Family Relations Satisfaction.* To assess the level of satisfaction in family relationships, the question “In general how satisfied are you with your Family Relationships?” was asked. An 8-point Likert scale ranging from “Extremely unhappy” to “Extremely happy” and including “Do not know” and “Prefer not to answer” made up the response choices.

*Ability to Confide in Others.* To assess whether participants have close relationships that provide an opportunity to connect on a deeper level to others, the question “How often are you able to confide in someone close to you?” was asked. An 8-point Likert scale ranging from “Never or almost never” to “Almost daily” and including “Do not know” and “Prefer not to answer” made up the response choices.

*Loneliness Satisfaction.* To assess the level of felt loneliness, the question “Do you often feel lonely?” was asked. The response choices were “Yes”, “No”, “Do not know” and “Prefer not to answer”.

*Financial Situation Satisfaction.* To assess the level of personal financial situation satisfaction, the question “In general, how satisfied are you with your Financial Situation?” was asked. An 8-point Likert scale ranging from “Extremely unhappy” to “Extremely happy” and including “Do not know” and “Prefer not to answer” made up the response choices.

*Leisure Activities/Hobbies.* To assess whether participants engage in leisure activities and regular hobbies, the question “Which of the following do you attend once a week or more often? (You can select more than one)” was asked. The response options were “Sports club or gym”, “Pub or social club”, “Religious group”, “Adult education class”, “Other group activity” and included “None of the above” and “Prefer not to answer”.

*Happiness*. To assess whether participants were currently happy in their life, the question “In general how happy are you” was asked. An 8-point Likert scale ranging from “Extremely Unhappy” to “Extremely Happy” and including “Do not know” and “Prefer not to answer” made up the response choices.

**Health Experience.** To assess health experience for both groups, two questions were asked: “In general how happy are you with your Health?” and “In general how satisfied are you with your Health?”. An 8-point Likert scale ranging from “Extremely unhappy” to “Extremely happy” and including “Do not know” and “Prefer not to answer” made up the response choices.

**Syndemic Contributors.** To include previously established Syndemic contributors within Syndemic models of health [26], two socio-political factors were included in the model, the *Townsend Deprivation Index* (TDI) and *ethnic background* (ethnicity). The TDI was calculated based on the postcode area of the participant and preceding national census output areas [50]. Ethnicity was derived from sequential branching questions from the initial assessment appointment via a touchscreen questionnaire [51].
